# Non-Syndromic Dentinogenesis Imperfecta Caused by Mild Mutations in *COL1A2*

**DOI:** 10.3390/jpm11060526

**Published:** 2021-06-08

**Authors:** Yejin Lee, Youn Jung Kim, Hong-Keun Hyun, Jae-Cheoun Lee, Zang Hee Lee, Jung-Wook Kim

**Affiliations:** 1 Department of Pediatric Dentistry, School of Dentistry & DRI, Seoul National University, Seoul 03080, Korea; lyj72255621@gmail.com (Y.L.); hege1@snu.ac.kr (H.-K.H.); 2 Department of Molecular Genetics, School of Dentistry & DRI, Seoul National University, Seoul 03080, Korea; ykim71@snu.ac.kr; 3 Seoul Chungdam Children’s Dental Center, Seoul 06072, Korea; childentist@hotmail.com; 4 Department of Cell and Developmental Biology, School of Dentistry & DRI, Seoul National University, Seoul 03080, Korea; zang1959@snu.ac.kr

**Keywords:** hereditary, mutational hotspot, dentinogenesis imperfecta, isolated dentin defect, tooth, discoloration, *COL1A2*

## Abstract

Hereditary dentin defects can be categorized as a syndromic form predominantly related to osteogenesis imperfecta (OI) or isolated forms without other non-oral phenotypes. Mutations in the gene encoding dentin sialophosphoprotein (DSPP) have been identified to cause dentinogenesis imperfecta (DGI) Types II and III and dentin dysplasia (DD) Type II. While DGI Type I is an OI-related syndromic phenotype caused mostly by monoallelic mutations in the genes encoding collagen type I alpha 1 chain (*COL1A1*) and collagen type I alpha 2 chain (*COL1A2*). In this study, we recruited families with non-syndromic dentin defects and performed candidate gene sequencing for *DSPP* exons and exon/intron boundaries. Three unrelated Korean families were further analyzed by whole-exome sequencing due to the lack of the *DSPP* mutation, and heterozygous *COL1A2* mutations were identified: c.3233G>A, p.(Gly1078Asp) in Family 1 and c.1171G>A, p.(Gly391Ser) in Family 2 and 3. Haplotype analysis revealed different disease alleles in Families 2 and 3, suggesting a mutational hotspot. We suggest expanding the molecular genetic etiology to include *COL1A2* for isolated dentin defects in addition to *DSPP*.

## 1. Introduction

Dentin is one of the major components comprising the teeth, and the crown part is covered by dental enamel, the hardest tissue in the human body. The root part inside the alveolar bone is covered by cementum, a thin bonelike tissue [1]. Dentin has similar mechanical properties with bone but has unique microstructures, parallel dentinal tubules from the dentin-enamel junction all the way to the pulp tissue [2]. Each dentinal tubule is filled with a long process of differentiated pulp cells, odontoblasts. This unique dentinal structure is believed to enable supporting extremely hard but brittle enamel and also providing a mechanosensory function to protect against noxious stimuli [3].

Hereditary dentin defects can occur with or without other syndromic phenotypes, like many other genetic diseases. Shields’ classification system consists of five types with two major categories: three types of dentinogenesis imperfecta (DGI) and two types of dentin dysplasia (DD) [4]. Among them, the DGI Type I (OMIM #166200) is the only syndromic form, a dental phenotype related to brittle bone diseases, osteogenesis imperfecta (OI). Some but not all OI patients exhibit variously discolored (brown to grey discoloration with opalescent hue) dentition and some characteristic tooth phenotypes, including bulbous crown, delayed dentin development or accelerated pulp chamber obliteration. The penetrance is not complete, and the expressivity is also variable among the same affected family members and even depends on the teeth in the dentition of an individual [5].

Mutations in the gene encoding dentin sialophosphoprotein (*DSPP*), the most abundant non-collagenous protein in the dentin matrix, have been identified to cause DGI Types II (OMIM #125490) [6,7], III (OMIM #125500) [8,9] and DD Type II (OMIM #125420) [10]. Now, these subtypes are recognized as allelic heterogeneity; therefore, there are some overlapping phenotypes because they are on a wide spectrum of the disorders depending on the severity of the mutation [11]. However, there is no other candidate gene except *DSPP* causing isolated DGI Types II and III and DD Type II.

Therefore, if a patient has a dental phenotype of DGI Types II or III or DD Type II without other non-oral symptoms, especially OI-related symptoms, the primary candidate gene to suspect is *DSPP*. Candidate gene sequencing of *DSPP* is not easy, especially for the last exon 5, which encodes part of the dentin sialoprotein (DSP) and the entire dentin phosphoprotein (DPP). Allele-specific cloning is necessary for the DPP-encoding region because it contains an ~2.4 kb repeat domain including an ~1.2 kb 3′ hypervariable repeat region [9]. Whole-exome sequencing seems to be not enough to get sufficient coverage and alignments in this region due to the extreme repeat.

In this study, we performed next-generation sequencing (NGS) analysis to identify the disease-causing mutations in three Korean families with an isolated hereditary dentin defect, whose *DSPP* gene candidate sequencing resulted in no disease-causing mutation. Genetic analysis identified mutations in the gene encoding collagen type I alpha 2 chain (*COL1A2*), a gene involved in OI pathogenesis.

## 2. Materials and Methods

### 2.1. Human Subject Enrollment

The study protocol and consent form were reviewed and approved by the Institutional Review Board of Seoul National University Dental Hospital. The nature of the study was explained, and informed consent was obtained from all participating individuals following the principles in the Declaration of Helsinki. Three unrelated nonconsanguineous Korean families were selected for this study.

### 2.2. Genomic DNA Isolation

A total of 3~4 mL of peripheral blood samples were collected, and genomic DNA was isolated by a conventional method with the NucleoSpin Blood L kit (Macherey-Nagel GmbH & Co., Düren, Germany) according to the manufacturer’s instructions. The quantity and quality of the DNA were quantitated by spectrophotometry measured by the OD_260_/OD_280_ ratio.

### 2.3. Candidate Gene Sequencing of the DSPP Gene

As a candidate gene approach, exons and exon-intron boundaries were amplified and sequenced as described before [12,13]. Briefly, PCR amplification and Sanger sequencing were performed for Exons 1, 2, 3 and 4 and the N-terminal end of Exon 5. Most of Exon 5, which is a highly repetitive and long DPP region, was amplified and cloned to sequence individual alleles. Multiple clones were sequenced and analyzed using Clustal Omega (https://www.ebi.ac.uk/Tools/msa/clustalo/ (accessed on 16 July 2019)).

### 2.4. Whole-Exome Sequencing

Candidate gene sequencing of the *DSPP* gene did not reveal a disease-causing variant. Whole-exome sequencing was performed for the selected individuals (III:2 in Family 1, III:2 in Family 2, and IV:2 in Family 3). Exomes were captured with the Agilent SureSelect^XT^ Human All Exon V6-Post Target Enrichment System, and 101-bp paired-end sequencing reads were generated with the NovaSeq sequencing platform (Macrogen, Seoul, Korea) (Table S1). A series of bioinformatic analyses was performed as previously described [14]. Briefly, sequencing reads were aligned to the reference human genome assembly hg38 using the Burrows-Wheeler Aligner alignment program, and then, the Samtools and Genome Analysis Tool Kit were used to get a list of small indels and single nucleotide changes [15,16,17]. Annotation of the variants was performed with Annovar against the single nucleotide polymorphism database (dbSNP) build 150 [18], and variants were filtered with the cut-off value of a minor allele frequency (MAF) of 0.01. The *COL1A2* mutations were confirmed by Sanger sequencing.

### 2.5. Haplotype Construction

Haplotypes of the affected individuals (III:2 in Family 2 and IV:2 in Family 3) were constructed using nearby whole-exome sequencing data to investigate whether they share the same disease allele from a common ancestor.

## 3. Results

### 3.1. Family 1

**Figure 1:**
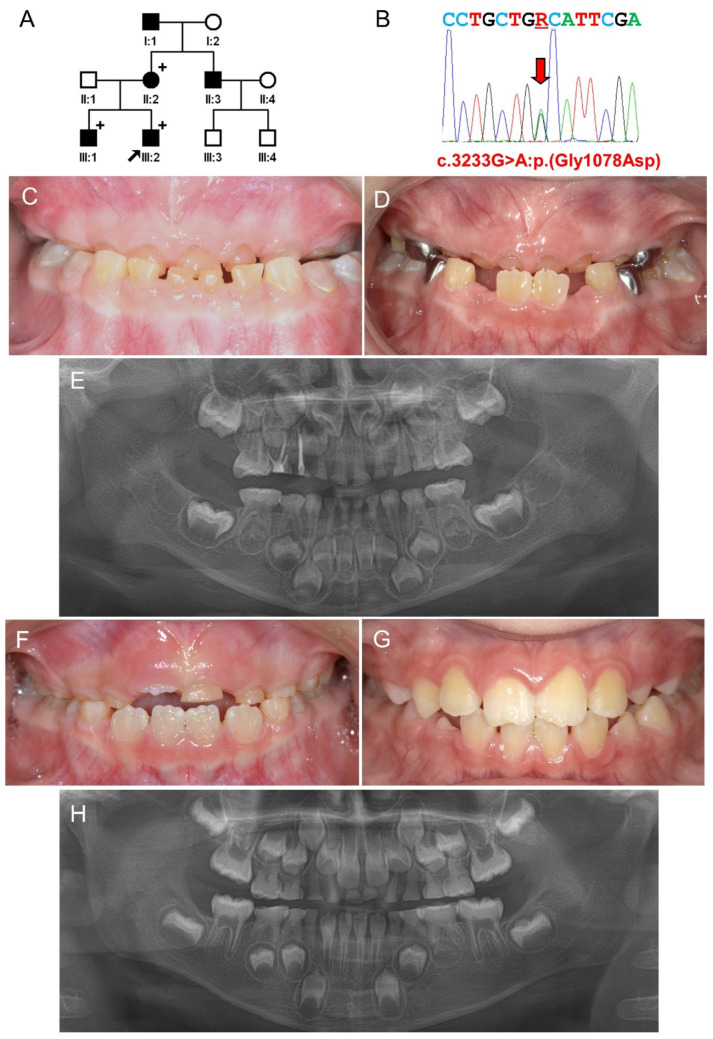
Pedigree, sequencing chromatogram, clinical photos, and panoramic radiograph of Family 1. (**A**) Pedigree of Family 1. Symbol filled black indicates affected individual, and the proband is indicated by a black arrow. Plus signs above the symbols indicate participating individuals. Individual ID is shown below the symbols. (**B**) Sequencing chromatogram of the proband. Nucleotide sequences are shown above the chromatogram. The red arrow indicates the location of the mutation (NM_000089.4:c.3233G>A) (R: A or G). (**C**) Frontal clinical photo of the proband at age 4 years. (**D**) Frontal clinical photo of the proband at age 6 years and 7 months. (**E**) Panoramic radiograph of the proband at age 4 years. (**F**) Frontal clinical photo of the proband’s brother at age 6 years. (**G**) Frontal clinical photo of the proband’s brother at age 8 years and 9 months. The incisal edges of the maxillary central incisors were fractured due to an accident. (**H**) Panoramic radiograph of the proband’s brother at age 6 years.

The proband was a four-year-old second son in a nonconsanguineous Korean family (Figure 1). He presented with complaints of excessive attrition and class III malocclusion. His deciduous dentition had a mild brown discoloration, especially in the anterior teeth. Severe attritions occurred in several teeth: the primary maxillary lateral incisors, primary maxillary canines, and primary maxillary first molars (Figure S1). Erupting mandibular central incisors also had a mild brown discoloration. His older brother also had a similar dental phenotype. His permanent anterior teeth exhibited minimal yellowish discoloration. Panoramic radiographs showed that the pulp chambers of the primary teeth were almost completely obliterated. The mother also had mild brown discoloration. Pulp chamber obliteration was seen in a panoramic radiograph. The grandfather and uncle of the proband were reported to have a similar dental phenotype. The family had no OI-related symptoms including fracture, articular pain, joint laxity, blue sclera, hearing problem, and hypodontia, and there was no other remarkable past medical history.

The filtered variants with the cut-off value of a MAF of 0.01 were further processed by removing variants with synonymous changes and non-frameshift deletions or insertions. After further manual checkup with Alamut Visual program (SOPHiA Genetics, Lausanne, Switzerland), whole-exome sequencing data analysis of the proband resulted in several candidate variants (Table S2). The candidate variant lists of Family 1 and Family 2 (Table S3) were compared to find a common disease-causing gene and resulted in *COL1A2* as a candidate gene. The mutation was a missense mutation caused by a transitional change of a glycine to an alanine (NM_000089.4:c.3233G>A) in Exon 48. This mutation would cause a change of glycine, which is a completely conserved amino acid in the helical domain, to aspartic acid at codon position 1078 (NP_000080.2:p.(Gly1078Asp)). Sanger sequencing confirmed the mutation in all participating affected individuals (Table S4, Figure S2). As mentioned above, the glycine at this position is completely conserved among the homologs of vertebrates (Figure S3). This mutation was predicted to be ‘possibly damaging’ by PolyPhen2 (probability of 1), ‘disease causing’ by mutation taster (with a score of 1.000) and combined annotation dependent depletion (CADD) resulted in a high (25.1) prediction value (Table S5). Interestingly, this mutation was listed in the dbSNP (rs72659332) without the allele frequency and was not listed in a public database, the genome aggregation database (GnomAD).

### 3.2. Family 2

**Figure 2:**
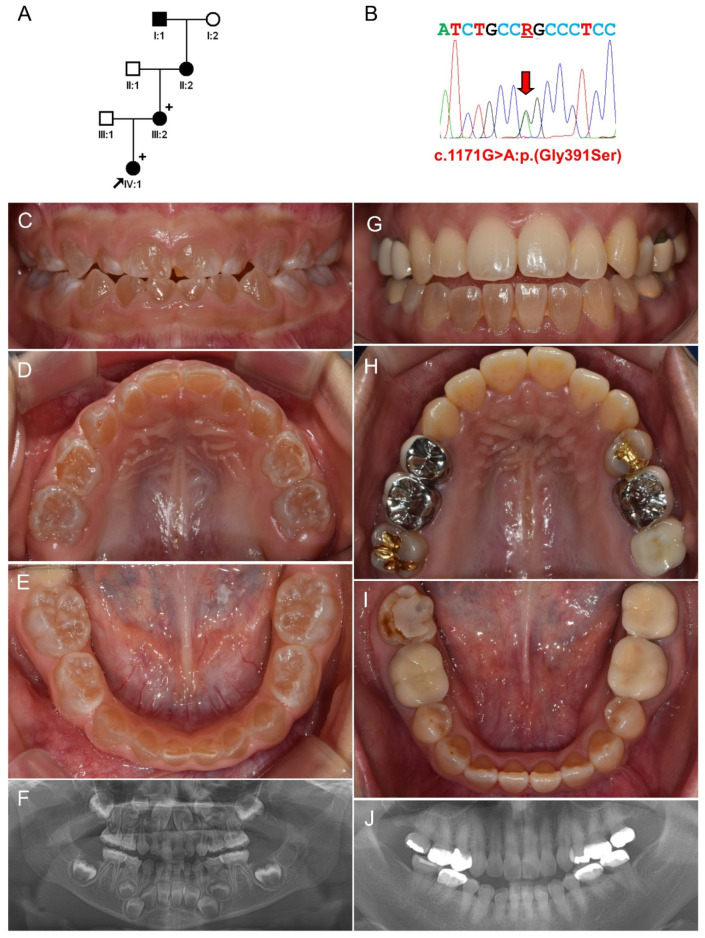
Pedigree, sequencing chromatogram, clinical photos, and panoramic radiograph of Family 2. (**A**) Pedigree of Family 2. The black filled symbol indicates the affected individual, and the proband is indicated by a black arrow. Plus signs above the symbols indicate participating individuals. (**B**) Sequencing chromatogram of the proband’s mother. Nucleotide sequences are shown above the chromatogram. The red arrow indicates the location of the mutation (NM_000089.4:c.1171G>A) (R: A or G). (**C***–***E**) Clinical photos of the proband at age 3 years and 10 months. (**F**) Panoramic radiograph of the proband at age 3 years and 10 months. (**G***–***I**) Clinical photos of the proband’s mother at age 32 years. Mild brown opalescent discoloration can be seen in the mandibular anterior teeth, but the maxillary anterior teeth are unremarkable. (**J**) Panoramic radiograph of the proband’s mother at age 32 years.

The proband was a three year ten month old daughter from a nonconsanguineous family and presented with a complaint of discolored teeth (Figure 2). She had a generalized brown discoloration in all deciduous dentition. Some teeth were not evenly discolored with some whitish areas. The mother also had a mild to moderate brown discoloration in the mandibular anterior teeth; however, the discoloration was minimal, if any, in the maxillary anterior teeth. The grandmother and great-grandfather were reported to have a similar dental phenotype. This family also had no OI-related symptoms listed above, and there was no other remarkable medical history.

Mutational screening of this family with Family 1 revealed a missense mutation in *COL1A2*, changing a conserved glycine to serine at codon 391 (NP_000080.2:p.(Gly391Ser)) by a transitional change of glycine to an alanine (NM_000089.4:c.1171G>A) in Exon 21. The mutation was confirmed by Sanger sequencing (Table S4), and the conservation among the homologs of vertebrates was also verified (Figure S3). This mutation was predicted as pathogenic by PolyPhen2 (possibly damaging; probability of 1), mutation taster (disease causing; a score of 1.000), and CADD (prediction value 33) (Table S5). This mutation was listed in the dbSNP (rs67707918); however, the allele frequency was not known and not listed in GnomAD.

### 3.3. Family 3

**Figure 3:**
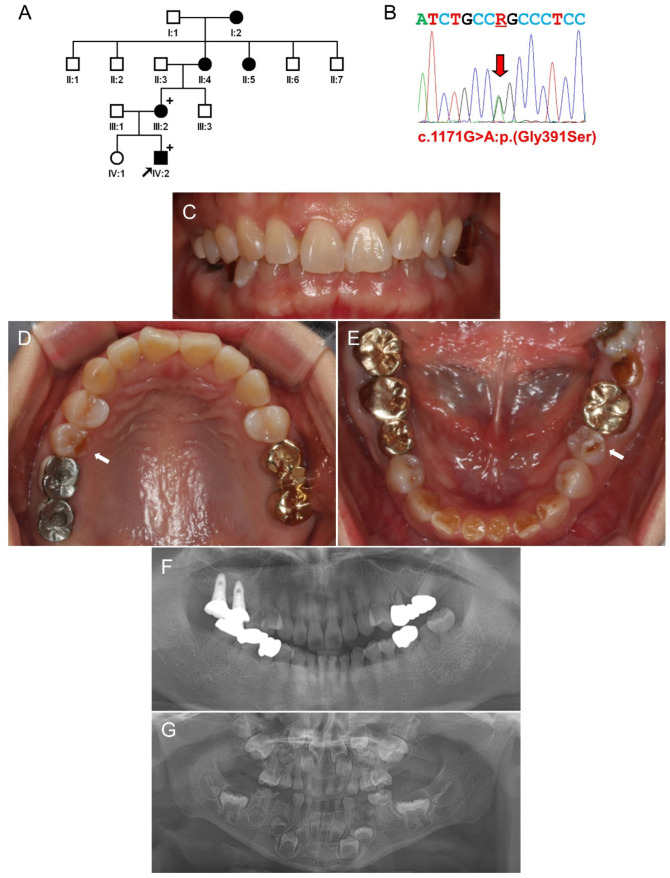
Pedigree, sequencing chromatogram, clinical photos, and panoramic radiograph of Family 3. (**A**) Pedigree of Family 3. The black filled symbol indicates the affected individual, and the proband is indicated by a black arrow. Plus signs above the symbols indicate participating individuals. (**B**) Sequencing chromatogram of the proband. Nucleotide sequences are shown above the chromatogram. The red arrow indicates the location of the mutation (NM_000089.4:c.1171G>A) (R: A or G). (**C**–**E**) Clinical photos of the proband’s mother at age 37 years. There are some attritions in the mandibular anterior teeth and premolars (white arrow); however, the maxillary anterior teeth look normal. (**F**) Panoramic radiograph of the proband’s mother at age 37 years. (**G**) Panoramic radiograph of the proband at age 4 years and 8 months.

The proband was a four year eight month old second son from a nonconsanguineous family (Figure 3). He was referred from a local dental clinic with a chief complaint of discolored and brittle teeth. Several mandibular primary molars fractured in combination with dental caries. The mother had mild discoloration and fractures in addition to the attrition in the mandibular anterior teeth and some premolars. An untreated mandibular left second molar exhibited exacerbated attrition and destruction. The grandmother and great-grandmother were reported to have a similar dental phenotype with mild discoloration; however, they did not have tooth fractures or severe attrition. There were no OI-related symptoms listed above, and there was no other remarkable medical history in this family as well.

**Figure 4:**
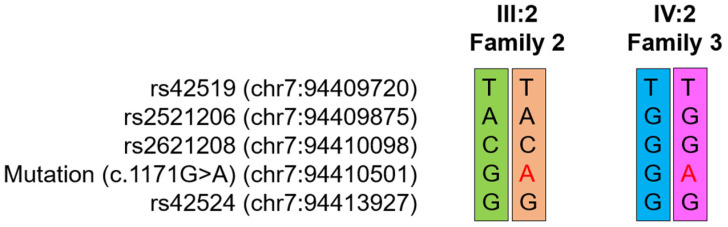
Haplotypes of individuals III:2 of Family 2 and IV:2 of Family 3. Markers are shown on the left with chromosomal locations. Different alleles are shown with different colors. Mutated nucleotides are indicated by a red color.

Interestingly, whole-exome sequencing data analysis revealed the same mutation with Family 2. The mutation in the affected family members was confirmed by Sanger sequencing (Figure S2). To determine whether this mutation is from a common ancestor, haplotype analysis was performed with nearby SNP sequences from the whole-exome sequencing. Comparison of the haplotypes between the affected individuals (III:2 of Family 2 and IV:2 of Family 3) showed that all the alleles are different indicating the disease-alleles are not identical by descent (Figure 4).

## 4. Discussion

The classic classification system of OI was proposed by Sillence et al. in 1979 and was based on clinical characteristics and inheritance pattern [19]: Type I is a nondeforming OI with little or no deformity, blue sclerae, hearing loss in 50% of families, and without (Subtype A) or with (Subtype B) DGI; Type II is perinatally lethal; Type III is progressive deforming, and Type IV is a moderate form, more severe than Type I but milder than Types II and III. With the advancement of genetic studies identifying more than 16 genes other than *COL1A1* or *COL1A2*, a modified classification was proposed to include genetic the etiology [20]. However, the most common mutations involved in OI are substitutions of glycine in the triple helix domain of Type I collagen [21].

The mutations identified in this study are listed in the dbSNP database build 130, which was updated on 30 April 2009. Both mutations were listed as unpublished data in the Supplementary Material from a study from a consortium of OI mutations in the helical domain of Type I collagen [22]: The p.(Gly1078Asp) was listed twice with the Type I and Type IV non-lethal phenotypes, and the p.(Gly391Ser) was listed with a Type III phenotype. Later on, the p.(Gly391Ser) was listed in a familial case with a Type IV phenotype in an OI-related paper [23]. These phenotypic variations could be a result of different genetic backgrounds and/or other genetic modifiers.

An interesting case with familial tooth agenesis and spontaneous DGI was reported to be caused by the maternal *PAX9* (c.43T>A, p.(Phe15Ile)) and de novo *COL1A2* (c.1171G>A, p.(Gly391Ser)) mutations, respectively [24]. The proband was a five-year-old boy of European descent and had no systemic signs and history of bone fracture. Bone radiographs showed no evidence supporting OI and were within normal limits. Recently, the same mutation was identified as an inherited one in a three-generation Thai family [25]. The affected individuals had DGI Type II phenotypes, such as discoloration, bulbous crowns, and dental pulp obliteration. However, they had normal facial features and hearing without blue sclerae, with no evidence of brittle bones and hyperflexible joints. The dental phenotype was variable among family members, and even within the same individual, some teeth (especially the maxillary anterior teeth and newly erupting mandibular anterior teeth) were unremarkable.

In this study, the same mutation was identified in two unrelated Korean families without OI-phenotype. Haplotype analysis revealed they did not share the same disease allele from a common ancestor. Taken together with a de novo occurrence in a European descent and a familial case in a Thai family, it is strongly suggested that this mutation is a mutational hotspot in *COL1A2*, related to the non-syndromic DGI.

When the OI classification was proposed, the genetic etiology was not known, and Types II and III were suspected to be autosomal recessive for some cases [19]. However, more recent reports have shown that these severe phenotypes are caused by a dominant-negative effect of the dominant *COL1A1* or *COL1A2* mutations, while true autosomal recessive inheritance is very rare [26]. The genotype-phenotype relationship revealed that the mutations in *COL1A2* are milder, predominantly nonlethal compared to the mutations in *COL1A1* [21].

Recently, a homozygous *COL1A2* mutation [c.604G>A, p.(Gly202Ser)] was reported in a Greek consanguineous family [27]. The proband and another affected sibling had severe skeletal fragility, but the mutation carriers were free from any OI features, even without DGI. Another homozygous *COL1A2* mutation [c.1009G>A, p.(Gly337Ser)] was identified in a Thai consanguineous family [28]. The proband, a 17-year-old woman, was diagnosed with OI Type III and DGI; however, her heterozygous carrier parents had only DGI.

It seems clear that mild and weak mutations in *COL1A2* can cause DGI as an isolated form, without OI-related symptoms. They share common phenotypic characteristics with variable expressivity depending on the teeth, unlike the DGI Types II and III phenotypes. In general, the severity of dentin defects caused by these mild *COL1A2* mutations seems to be worse than the DD Type II but not as severe as DGI Type II.

In conclusion, we recruited three non-syndromic Korean families with hereditary dentin defects, and a mutational study including candidate sequencing of *DSPP* and whole-exome sequencing revealed heterozygous mutations in *COL1A2*. Therefore, we suggest including *COL1A2* as a candidate gene for isolated dentin defects in addition to *DSPP*.

## Data Availability

The data presented in this study are openly available in ClinVar (http://www.ncbi.nlm.nih.gov/clinvar (accessed on 18 May 2021)), Submission ID: SUB9674669 and SUB9674675.

## Supplementary Materials

The following are available online at https://www.mdpi.com/article/10.3390/jpm11060526/s1, Table S1: statistics for exome sequencing; Table S2: filtered sequence variants of Family 1; Table S3: filtered sequence variants of Family 2; Table S4: *COL1A2* PCR primers; Table S5: variants identified in *COL1A2* gene (GenBank: NM_000089.4); Figure S1: clinical photos and panoramic radiograph of Family 1; Figure S2: sequencing chromatograms; Figure S3: sequence alignments of vertebrate orthologs.
