# Supplementary material for: Non-Syndromic Dentinogenesis Imperfecta Caused by Mild Mutations in COL1A2
Source: J Pers Med. 2021 Jun 8;11(6):526. doi: 10.3390/jpm11060526 (PMC8229930; doi:10.3390/jpm11060526)
Supplement: Supplementary file 1 [file jpm-11-00526-s001.zip › jpm-1244920-supplementary.pdf]

Non-Syndromic Dentinogenesis Imperfecta Caused by Mild Mutations in *COL1A2*

Yejin Lee [1], Youn Jung Kim [2], Hong-Keun Hyun [1], Jae-Cheoun Lee [3], Zang Hee Lee [4], Jung-Wook Kim [1,2,*]

[1]Department of Pediatric Dentistry
School of Dentistry & DRI, Seoul National University, Seoul, Korea.
[2]Department of Molecular Genetics
School of Dentistry & DRI, Seoul National University, Seoul, Korea.
[3]Seoul Chungdam Children's Dental Center, Seoul, Korea.
[4]Department of Cell and Developmental Biology
School of Dentistry & DRI, Seoul National University, Seoul, Korea.

Table S1. Statistics for exome sequencing.

Sample		Total reads	Mapping rate (%)	Median target coverage	Coverage of target region (%)	Fraction of target covered with at least	
						20X	10X
Family 1	III:2	68,336,428	99.9	53	96.4	88.9	94.4
Family 2	III:2	68,251,167	99.9	52	96.3	89.0	94.4
Family 3	IV:2	67,482,642	99.9	47	96.4	85.3	93.7

Table S2. Filtered sequence variants of family 1.

Genomic Variant	Gene	Changes	dbSNP150	CADD
chr1:22912473T>C	EPHB2	NM_017449:c.T2726C:p.I909T		16.1
chr2:69844171A>T	GMCL1	NM_178439:c.A733T:p.N245Y		22.5
chr6:38938141C>T	DNAH8	NM_001371:c.C11080T:p.L3694F		31.0
chr6:56170661G>T	COL21A1	NM_030820:c.C1014A:p.N338K	rs187410130	1.7
chr7:94427261G>A	COL1A2	NM_000089:c.G3233A:p.G1078D	rs72659332	25.1
chr7:100431151G>A	MEPCE	NM_019606:c.G1133A:p.G378D		13.7
chr9:113497405G>A	RGS3	NM_144488:c.841+1G>A		22.7
chr13:19178254G>A	TUBA3C	NM_006001:c.C367T:p.R123C	rs201563264	32.0

Combined Annotation Dependent Depletion (CADD), http://cadd.gs.washington.edu

Table S3. Filtered sequence variants of family 2.

Genomic Variant	Gene	Changes	dbSNP150	CADD
chr1:85745509G>T	COL24A1	NM_152890:c.4438-3C>A		
chr2:67404090TCAAAdel	ETAA1	NM_019002:c.1408_1412del:p.S470Qfs*7		
chr3:48579635G>A	COL7A1	NM_000094:c.C5188T:p.R1730X	rs746053763	38.0
chr7:94410501G>A	COL1A2	NM_000089:c.G1171A:p.G391S	rs67707918	33.0
chr14:104932816insT	PLD4	NM_138790:c.1373dupT:p.L458Ffs*25		
chr16:1351521C>A	TSR3	NM_001001410:c.G190T:p.E64X		37.0
chr17:9917317G>A	GAS7	NM_003644:c.C922T:p.L308F		24.6

Combined Annotation Dependent Depletion (CADD), http://cadd.gs.washington.edu

Table S4. *COL1A2* PCR primers.

Primer name	Sequences	PCR size (bp)
hCOL1A2x20-21-F	5'-TTCTCTTTACCTTGACCCACAAA-3'	455
hCOL1A2x20-21-R	5'-CAATTCCCAGTGAAGGGGTA-3'	
hCOL1A2x47-48-F	5'-TGTCTCTTGACATGTGCTCTGA-3'	414
hCOL1A2x47-48-R	5'-TGTCTTGGTTTAGTCTGAAGAAAA-3'	

Table S5. Variants identified in *COL1A2* gene (GenBank: NM_000089.4).

Location	Variant	Protein	CADD	PolyPhen2	Mutation Taster	dbSNP created	Alleles gnomAD
Exon 21	c.1171G>A	p.Gly391Ser	33	Possibly damaging (1)	Disease causing (1)	rs67707918 dbSNP130	N/A
Exon 48	c.3233G>A	p.Gly1078Asp	25.1	Possibly damaging (1)	Disease causing (1)	rs72659332 dbSNP130	N/A

Combined Annotation Dependent Depletion (CADD), http://cadd.gs.washington.edu
PolyPhen2, http://genetics.bwh.harvard.edu/pph2/
Mutation Taster, http://www.mutationtaster.org/
Genome aggregation database, https://gnomad.broadinstitute.org/
dbSNP, http://www.ncbi.nlm.nih.gov/projects/SNP/

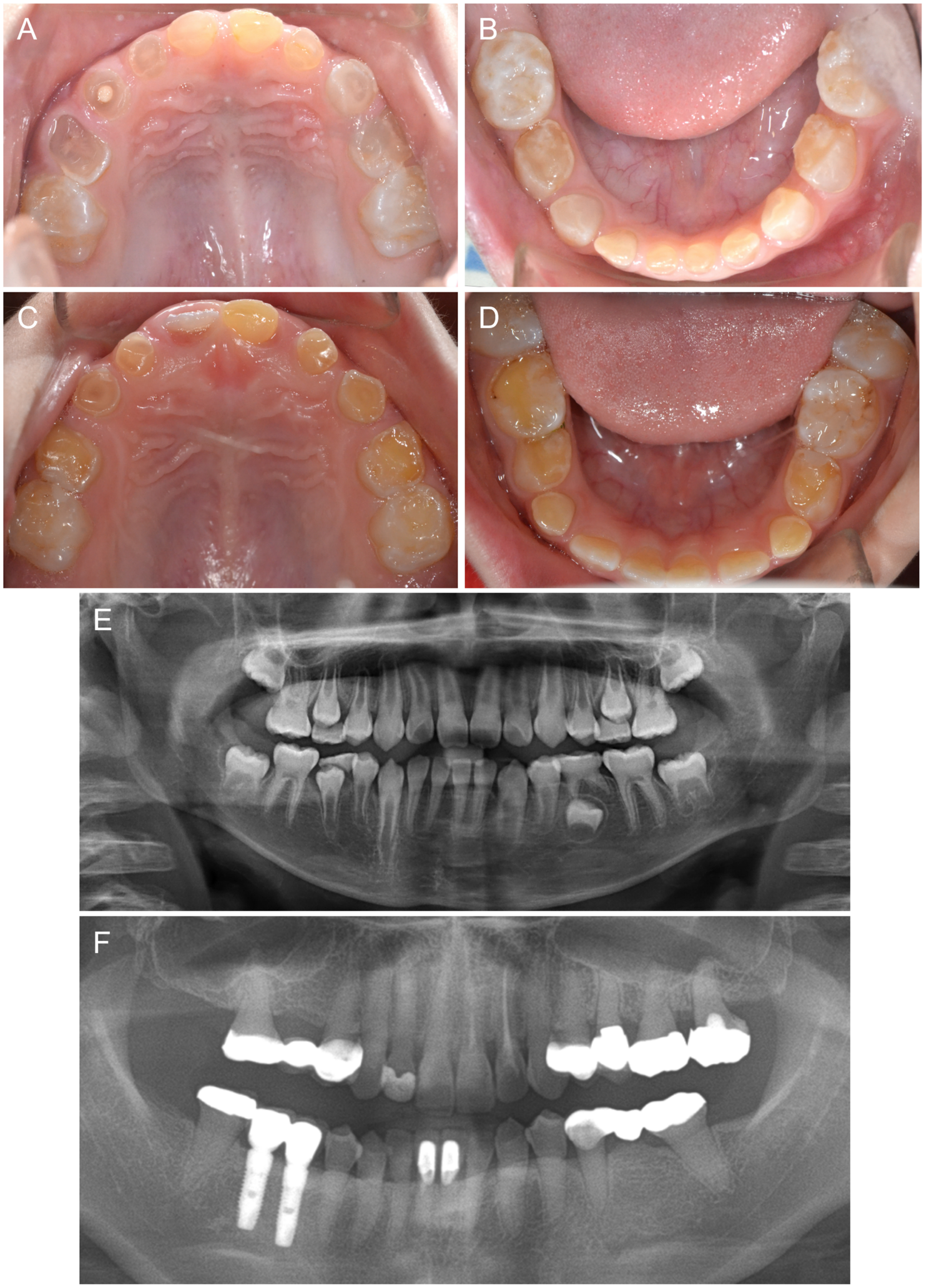

Figure S1. Clinical photos and panoramic radiograph of family 1. (**A, B**) Maxillary and mandibular clinical photos of the proband at age 4 years. (**C, D**) Maxillary and mandibular clinical photos of the proband's brother at age 6 years. (**E**) Panoramic radiograph of the proband's brother at age 8 years 11 months. (**F**) Panoramic radiograph of the proband's mother at age 36 years.

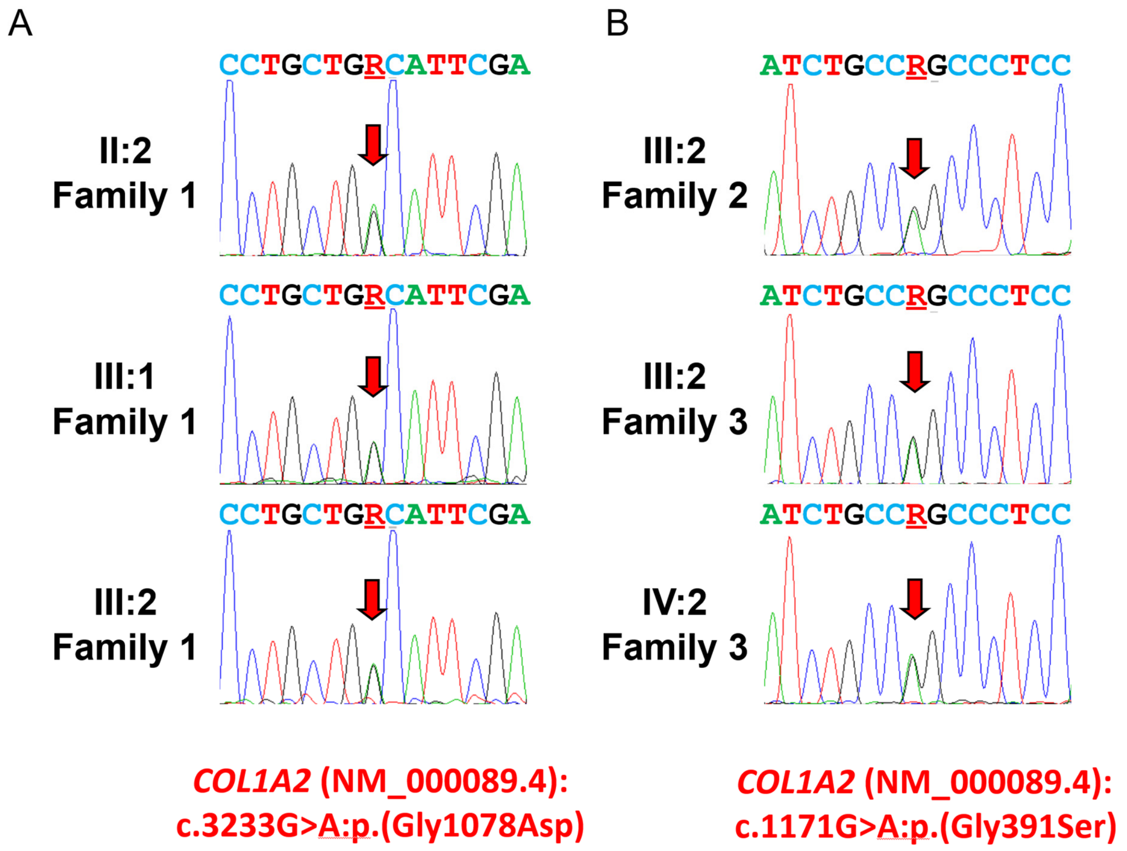

Figure S2. Sequencing chromatograms. (**A**) Sequencing chromatograms of family 1. Individual IDs are shown on the left. (**B**) Sequencing chromatograms of family 2 and 3. Individual IDs are shown on the left.

```
                      Gly391         Gly1078
                        ↓               ↓
COL1A2_Human       EAGSAGPPGPP     TVGPAGIRGPQ
COL1A2_Chimpanzee  EAGSAGPPGPP     TVGPAGIRGPQ
COL1A2_Monkey      EVGSAGPPGPP     TVGPAGIRGPQ
COL1A2_Dog         EAGSAGPSGPP     TVGPAGIRGSQ
COL1A2_Cattle      EIGPAGPPGPP     AVGPAGIRGSQ
COL1A2_Mouse       EAGSAGPAGPP     PVGPAGVRGSQ
COL1A2_Rat         EPGSAGPAGPP     PVGPAGVRGSQ
COL1A2_Chicken     EPGSAGPPGPA     PIGPAGVRGSH
COL1A2_Frog        EAGSSGPAGNA     PIGPVGLRGPA
COL1A2_Zebrafish   EQGPTGPLGLR     AIGPPGHRGPA
                   * *  ** *         ** * **
```

Figure S3. Sequence alignments of vertebrate orthologs. Amino acid sequences from vertebrates are aligned to determine the conservation. Conserved amino acids are indicated with * under the sequence. The mutated amino acids are indicated by red arrows.
